# Non-Adherence to Statin Treatment in Older Patients with Peripheral Arterial Disease Depending on Persistence Status

**DOI:** 10.3390/biomedicines8100378

**Published:** 2020-09-25

**Authors:** Martin Wawruch, Gejza Wimmer, Jan Murin, Martina Paduchova, Miriam Petrova, Tomas Tesar, Petra Matalova, Beata Havelkova, Michal Trnka, Emma Aarnio

**Affiliations:** 1 Institute of Pharmacology and Clinical Pharmacology, Faculty of Medicine, Comenius University, 811 08 Bratislava, Slovakia; miriampetrova1@gmail.com; 2 Institute of Measurement Science, Slovak Academy of Sciences, 841 04 Bratislava, Slovakia; dzibo7@gmail.com; 3 1st Department of Internal Medicine, Faculty of Medicine, Comenius University, 813 69 Bratislava, Slovakia; jan.murin@gmail.com; 4 Department of Angiology, Health Centre, 917 01 Trnava, Slovakia; martina.paduchova@medena.sk; 5 Department of Organisation and Management of Pharmacy, Faculty of Pharmacy, Comenius University, 832 32 Bratislava, Slovakia; 6 Department of Pharmacology, Faculty of Medicine and Dentistry, Palacky University, 775 15 Olomouc, Czech Republic; petra.matalova@fnol.cz; 7 General Health Insurance Company, 851 04 Bratislava, Slovakia; beata.havelkova@vszp.sk; 8 Institute of Medical Physics, Biophysics, Informatics and Telemedicine, Faculty of Medicine, Comenius University, 813 72 Bratislava, Slovakia; michal.trnka@fmed.uniba.sk; 9 Institute of Biomedicine, University of Turku, 20014 Turku, Finland; emma.aarnio@uef.fi; 10 School of Pharmacy, University of Eastern Finland, 70211 Kuopio, Finland

**Keywords:** non-adherence, peripheral arterial disease, statins, polypharmacy, risk factors

## Abstract

The effectiveness of statins in secondary prevention of peripheral arterial disease (PAD) largely depends on patients’ adherence to treatment. The aims of our study were: (a) to analyze non-adherence during the whole follow-up in persistent patients, and only during persistence for non-persistent patients; (b) to identify factors associated with non-adherence separately among persistent and non-persistent patients. A cohort of 8330 statin users aged ≥65 years, in whom PAD was newly diagnosed between January 2012–December 2012, included 5353 patients persistent with statin treatment, and 2977 subjects who became non-persistent during the 5-year follow-up. Non-adherence was defined using the proportion of days covered <80%. Patient- and statin-related characteristics associated with non-adherence were identified with binary logistic regression. A significantly higher proportion of non-adherent patients was found among non-persistent patients compared to persistent subjects (43.6% vs. 29.6%; *p* < 0.001). Associated with non-adherence in both persistent and non-persistent patients was high intensity statin treatment, while in non-persistent patients, it was employment and increasing number of medications. In patients with a poor adherence during their persistent period, an increased risk for discontinuation may be expected. However, there is also non-adherence among persistent patients. There are differences in factors associated with non-adherence depending on patients’ persistence.

## 1. Introduction

Peripheral arterial disease (PAD) mostly represents a local manifestation of a systemic disease–atherosclerosis. The overall prevalence of PAD is in the range of 3–10% and it increases to 15–20% in subjects older than 70 years. PAD is associated with an annual mortality rate of 4–6% [1,2]. In our study, PAD refers to ischemic disease of the lower limbs.

The treatment of PAD is aimed at reducing the risk of major cardiovascular (CV) events (myocardial infarction (MI), ischemic stroke, and CV death), the risk of major adverse limb events (amputations and acute limb ischemia), improvement of limb symptoms and functional status. Besides lifestyle modifications (smoking cessation, dietary changes, and weight loss), it includes the treatment of modifiable risk factors (control of blood pressure, blood glucose, and lipid concentrations), and administration of antiplatelet agents, angiotensin-converting enzyme inhibitors and statins in high doses regardless of the patient’s serum cholesterol level [1,3,4,5,6]. Statin treatment reduces not only the risk of adverse CV events, but it has also beneficial effects on the limb prognosis. According to the analysis by Kumbhani et al. [7], statin use was associated with an ~18% lower rate of adverse limb outcomes (worsening symptoms, peripheral revascularisation, and ischemic amputations). Mohler 3rd et al. [8] reported an improvement in pain-free walking distance and community-based physical activity in patients with intermittent claudication after 12 months of treatment with atorvastatin.

The effectiveness of statins in secondary PAD prevention largely depends on patients´ adherence to treatment. Adherence includes three interrelated yet distinct phases: initiation, implementation, and persistence. Initiation represents a beginning of the process when a patient takes the first dose of a prescribed medication. Implementation reflects the extent to which a patient´s dosing regimen corresponds to that prescribed by a physician. Persistence represents the length of time between initiation and the last dose which precedes discontinuation [9,10]. Our previous study [11] was focused on the analysis of non-persistence with statin treatment in the group of 8330 PAD patients aged ≥65 years. During the 5-year follow-up, non-persistence was recorded in 2977 (37.5%) subjects. Factors associated with the likelihood of non-persistence were identified.

Analysis of adherence using drug dispensation data available in databases of health insurance companies represents one of many possible methods of adherence measurement. These databases make it possible to analyze implementation using indexes like proportion of days covered (PDC) or medication possession ratio (MPR). Both indexes represent validated measures commonly used in adherence studies. Patients with values of these indexes ≥80% are generally accepted as adherent to their treatment [12,13,14,15,16,17,18]. Many studies have evaluated adherence using PDC/MPR without differentiation between the groups of persistent and non-persistent patients [15,16]. However, there are some studies which calculated MRP with regard to persistent/non-persistent status in groups of patients with statin or antihypertensive treatment [17,18]. Many studies evaluating statin non-adherence in the general population of statin users and several studies analyzing this issue in patients after MI or stroke/transient ischemic attack have been published [15,16,19,20,21,22]. However, to the best of our knowledge, despite the importance of adherence to statins in terms of the reduction of CV risk and improvement of limb prognosis, there are no studies analyzing statin non-adherence specifically in the group of older PAD patients. The aims of our study were: a) to analyze non-adherence identified according to PDC < 80% separately in the group of patients who were persistent during the whole follow-up, and to perform this analysis only within the persistent period in patients who became non-persistent with statin treatment during follow-up; b) to identify factors associated with the patient’s probability of non-adherence separately in the groups of persistent and non-persistent patients.

## 2. Experimental Section

### 2.1. Database and Study Population

For the register-based retrospective cohort study described in this manuscript, a cohort of 8330 statin users aged ≥65 years, in whom PAD was newly diagnosed between 1 January and 31 December 2012, was used. This cohort was analyzed in our previous manuscript focused on the analysis of non-persistence with statin treatment [11]. The selection of patients for the study cohort is described in detail in that manuscript. In Slovakia, there are three health insurance providers (1 state and 2 private companies) and all employed citizens have to be insured. The database of the General Health Insurance Company (guaranteed by the state) represented a source of data for our study. This company is the largest health insurance provider in the Slovak Republic, covering approximately 63% of the population. The study cohort (*n* = 8330) included 5353 patients persistent with statin treatment and 2977 subjects who became non-persistent during the 5-year follow-up period. In our previous study, non-persistence was identified according to the treatment gap defined as a 6-month period without statin prescription observed after the estimated date of the last day covered by the last package of prescribed medication. In this study, non-persistent subjects were more likely to be female, new statin users, those who started treatment with atorvastatin or rosuvastatin, and those who had a higher co-payment. On the other hand, persistent subjects were more likely to be older, had a history of ischemic stroke or had diabetes mellitus, had a general practitioner as index prescriber, had a higher overall number of medications, and were using certain CV co-medications (cardiac glycosides, loop diuretics, and mineralocorticoid receptor antagonists) [11].

According to the legal principles of our country, the study did not require approval of an Ethical Committee. The rules of personal data protection were fully respected. Only de-identified anonymized data were available for our research. We had no direct contact with patients as all information was register-based. The General Health Insurance Company granted us permission to use data collected from their database.

### 2.2. Analysis of Non-Adherence

Non-adherence was evaluated using the PDC index [14]. PDC was calculated by dividing the number of days covered by statin treatment by the number of days of the follow-up period during which a patient was persistent with this treatment. It means that, in persistent patients, the number of days of their whole follow-up was used as a denominator of the equation, whereas in non-persistent patients, only the number of days of their period of persistence (not including the 6-month treatment gap period) represented a denominator. In the group of non-persistent patients, the use of the number of days of their whole follow-up as a denominator of the equation would lead to overestimation of their real non-adherence. In these analyses, the dosage of 1 tablet of statin per day was assumed [23]. If a statin was dispensed before the end of the previous supply, the use of the new supply was considered to start after the previous supply had been completely depleted. Non-adherence was defined using the threshold of PDC < 80% [24].

### 2.3. Factors Associated with Non-Adherence

The same patient- and statin-related characteristics as those evaluated in our previous study focused on the analysis of non-persistence [11] were analyzed as factors potentially associated with non-adherence in the study described in this manuscript. Data on these characteristics were collected at the time of inclusion in the study. The only exception were the data on the history of CV events (MI, stroke, and transient ischemic attack) which were recorded within a period of 5 years before the index date of the study described in our previous manuscript (i.e., the date of the first dispensation of statin after the PAD diagnosis) [11]. Criteria for the evaluation of statin intensity were also described in detail in our previous manuscript [11].

### 2.4. Statistical Analysis

Continuous variables were characterized as medians [interquartile ranges], while categorical variables were characterized as frequencies and percentages. To compare categorical variables between the two groups, the chi-square test was applied. The normality of the distribution of continuous variables was analyzed by the Kolmogorov–Smirnov test. This test revealed significant differences between the Gaussian distribution and distribution of data in all analyzed groups (*p* < 0.001). Because of the non-Gaussian distribution of evaluated variables, we applied the non-parametric Mann–Whitney U test to compare continuous variables between the two groups.

The most important patient- and statin-related characteristics associated with non-adherence were identified in the multivariate analysis using the binary logistic regression model. Odds ratios and corresponding 95% confidence intervals were calculated for each characteristic [25].

All statistical tests were carried out at a significance level of α = 0.05. The statistical software IBM SPSS for Windows, version 26, was used (IBM SPSS Inc., Armonk, NY, USA).

### 2.5. Sensitivity Analyses

Since the 80% threshold defining non-adherence was selected empirically [24], sensitivity analyses using different thresholds (50%, 60%, 70%, and 90%) were aimed at analyzing whether lower or higher thresholds may influence the results of the logistic regression models. The 5-year follow-up represents a relatively long follow-up period. Since adherence may vary over time, a sensitivity analysis using a shorter 3-year follow-up was performed.

## 3. Results

The baseline characteristics of the study cohort have been reported in our previous manuscript [11], and those characterizing adherent and non-adherent patients are shown in Table 1.

The PDC was significantly higher in the group of persistent patients compared to that of non-persistent subjects (median [interquartile range]: 92.3% [24.3] vs. 84.9% [35.1]; *p* < 0.001 according to the Mann–Whitney U test). Non-adherent patients (PDC < 80%) constituted a larger proportion in the group of non-persistent patients (43.6% of *n* = 2977) compared to that of persistent subjects (29.6% of *n* = 5353) (*p* < 0.001 according to the χ^2^-test) (Table 1).

The results of the multivariate analysis of factors potentially influencing the patient´s likelihood of non-adherence are shown in Table 2. In the model which analyzed these factors in the group of persistent patients, atorvastatin, being a new statin user (patient in whom statin treatment was initiated in association with PAD diagnosis), and increasing patient´s co-payment were associated with better adherence, while high intensity statin treatment was associated with non-adherence. In the model that analyzed risk factors of non-adherence in the group of non-persistent patients, increasing age, dementia, atorvastatin, cardiac glycosides, and beta-blockers were associated with better adherence, whereas employment, high intensity statin treatment, and increasing number of medications were associated with non-adherence.

### Sensitivity Analyses

In the case of the use of different PDC thresholds to define non-adherence (50%, 60%, 70%, and 90%), the following proportions of non-adherent patients were found in the groups of persistent and non-persistent patients: 5.1% and 9.6%; 11.1% and 19.4%; 19.0% and 31.0%; 45.5% and 55.4%, respectively. When using the shorter 3-year follow-up period, non-adherent patients (PDC < 80%) represented 31.6% and 44.5% of the groups of persistent and non-persistent patients, respectively. The results of the multivariate analyses of factors potentially influencing the patient´s risk for non-adherence evaluated in models using different thresholds defining non-adherence (50%, 60%, 70%, and 90%) are shown in Supplementary Table S1. The results of the logistic regression models with shorter 3-year follow-up period and with standard 80% threshold defining non-adherence are summarized in Supplementary Table S2. There are differences in factors associated with non-adherence among particular logistic regression models, but atorvastatin (in persistent and non-persistent patients) and being a new statin user (in persistent patients) were consistently associated with better adherence, whereas high intensity statin treatment (in persistent patients) was consistently associated with non-adherence in all models presented in Table 2 and Supplementary Tables S1 and S2.

## 4. Discussion

Our study was focused on the analysis and comparison of non-adherence between the groups of older PAD patients persistent or non-persistent with statin treatment during a 5-year follow-up as well as on identifying factors associated with non-adherence in both groups. A significantly higher proportion of non-adherent patients was found in the group of non-persistent patients compared to that of persistent subjects (43.6% vs. 29.6%). This result indicates an insufficient medication-taking behaviour in non-persistent patients occurring already during their persistent period preceding discontinuation. These patients represent a risky group in terms of both phases of the adherence process: implementation and persistence [9,10]. Our logistic regression model performed in the group of non-persistent patients revealed more factors associated with non-adherence than that analyzing this issue in persistent patients. Factors associated with adherence and non-adherence largely differed between groups of persistent and non-persistent patients. High intensity statin treatment and administration of atorvastatin represented the only factors which were consistently associated with non-adherence in both groups. To the best of our knowledge, there are no similar studies focused on statin non-adherence specifically in older PAD patients. For this reason, studies cited in the text below analyzed mostly the general population of statin users.

Among socio-demographic characteristics, increasing age was associated with better adherence, but only in the group of non-persistent patients. In our previous study, increasing age represented a factor decreasing the likelihood of non-persistence [11]. It seems that a relatively younger age is associated with shorter persistence and, in addition, also worse adherence prior to non-persistence. In line with our results, the relative risk for adherence characterized by MPR ≥ 80% was significantly elevated for older individuals (aged 65+ years) in the study by Warren et al. [26]. In the study by Alfian et al. [17], younger age (<50 years) represented a predictor of non-adherence (MPR < 80%) in persistent patients. In this study, no association was found between non-adherence in persistent patients and the number of other medications dispensed, medication dispensed before statin initiation, and dose of initiation statin. On the other hand, in our present study, employment appeared as a factor increasing the non-persistent patients’ risk for non-adherence. In line with our finding, Warren et al. [26] also reported more non-adherence found in patients who were employed.

Among comorbid conditions, dementia represented a factor associated with better adherence in the group of non-persistent patients. Adherence of such patients may appear as good in case when their family members or caregivers administer properly all prescribed medications. In contrast to our finding, Ofori-Asenso et al. [27] reported low adherence and high discontinuation of statin treatment among older Australian patients with dementia, which may have been due to intentional cessation.

Increasing the number of medications appeared as a factor increasing the probability of non-adherence only in the group of non-persistent patients. However, in our previous study, increasing the number of medications represented a factor associated with a lower likelihood of non-persistence [11]. These results may indicate that in patients with polypharmacy who do not adhere sufficiently to statin treatment, an increased risk for discontinuation may be expected after a certain period of persistence. Polypharmacy was identified as a factor associated with non-adherence in the systematic review by Gellad et al. [28]. In contrast to our results, polypharmacy, identified as a concurrent use of 5 or more drugs, represented a factor associated with a lower probability of non-adherence in the retrospective cohort study by Ofori-Asenso et al. [29] which analyzed predictors of first-year non-adherence and discontinuation among older adults.

In our study, being a new statin user, in whom statin treatment was initiated in association with PAD diagnosis, was associated with better adherence only in the group of persistent patients. On the other hand, new statin users were at increased risk for non-persistence in our previous study [11]. These results suggest that new users may incline to discontinuation, e.g., in case of adverse drug reactions occurring early after statin treatment initiation. However, if they persist on treatment, their adherence seems to be good. This good adherence may be associated with a proper physician explanation of the importance of statin treatment at the time of its initiation because of newly diagnosed PAD. In contrast to our results, being a new statin user represented a variable associated with non-adherence in the meta-analysis by Lemstra et al. [30].

Administration of atorvastatin was associated with better adherence to statin treatment in both groups of persistent and non-persistent patients, whereas administration of cardiac glycosides and beta-blockers was associated with better adherence only in the group of non-persistent patients. The design of our study does not make it possible to identify the reasons for these findings. In line with our results, administration of atorvastatin and the use of CV medications represented a significant predictor of adherence in the study by Aarnio et al. [15] that analyzed register-based predictors of adherence among new statin users in Finland.

High intensity statin treatment represented a factor associated with non-adherence in both groups of persistent and non-persistent patients. However, it represented a factor associated with a decreased probability of non-persistence in our previous study [11]. These results may suggest that although patients receiving high intensity statin treatment are aware of the necessity of continuous long-term administration of statins in secondary prevention of PAD, they may have a certain fear of taking higher doses of statins because of a higher risk of adverse drug reactions. This fear may be a reason for non-adherence [31], such as skipping doses or taking lower doses.

Higher co-payment appeared as a factor associated with better adherence in the group of persistent patients in our present study, whereas it represented a factor associated with an increased risk for non-persistence in our previous study [11]. In contrast to our findings, increased co-payment represented a factor associated with an increased statin non-adherence in the systematic review and meta-analysis by Ofori-Asenso et al. [32] focused on factors associated with non-adherence and discontinuation of statin treatment among older patients aged ≥65 years. More research is needed to see whether the association between adherence and co-payment differs between persistent and non-persistent patients also in other patients and study groups.

Our study has some limitations which should be taken into consideration when the results of the study are interpreted. The database of the General Health Insurance Company, which served as a source of data for our study, was compiled for insurance and not research purposes. This database does not make it possible to determine whether dispensed medications were taken properly according to physicians´ recommendations. Our analysis using PDC represents only an indirect estimation of the real adherence. Another limitation is represented by the impossibility of calculation of the Charlson comorbidity index since information about some diseases necessary for calculation of this index may not be sufficiently captured in our database [33]. For example, data about chronic kidney disease may be underreported since, in such patients, medications are frequently prescribed for treatment of accompanying comorbid conditions or complications (e.g., arterial hypertension, diabetes mellitus). Consequently, codes of these accompanying conditions or complications are available on doctors´ prescriptions, which represented a source of data for the database of the General Health Insurance Company. Neither did our database include information about surgical or interventional treatment which may also influence adherence to statins. On the other hand, the large sample size, which covers all administrative regions of the Slovak Republic and detailed data on dispensation of statins and patient-related characteristics, represent the strength of our study.

Despite the limitations mentioned above, our study revealed significant differences in the proportions of non-adherent patients as well as in factors associated with non-adherence between the groups of persistent and non-persistent patients. This encourages future studies also to differentiate between persistent patients and patients ultimately becoming non-persistent. Patient- and drug-related characteristics associated with non-adherence determined in this study may be helpful for identifying groups of patients who are at increased risk for non-adherence. Younger patients, employed subjects, those with a higher number of medications taken concurrently, and those receiving high intensity statin treatment, represented groups of patients with an increased risk for non-adherence.

## 5. Conclusions

A significantly higher proportion of non-adherent patients in the group of non-persistent patients compared to persistent subjects indicates a close relationship between particular phases of the adherence process, namely implementation and persistence [9,10]. In patients with a poor adherence during their persistent period, an increased risk for discontinuation may be expected. Patient- and drug-related characteristics associated with non-adherence may serve for identification of PAD patients to whom special educational efforts aimed at the improvement of their adherence to statins indicated in the secondary PAD prevention should be targeted.

## Figures and Tables

**Table 1:** Baseline characteristics of the study cohort.

	Persistent Patients (*n* = 5353)			Non-Persistent Patients (*n* = 2977)		
	Adherent (*n* = 3769)	Non-Adherent (*n* = 1584)	*p*	Adherent (*n* = 1678)	Non-Adherent (*n* = 1299)	*p*
*Socio-demographic characteristics*						
Age	74.0 [10.0]	74.0 [10.0]	0.673 *	72.0 [9.0]	72.0 [8.0]	0.040 *
Female sex	2040 (54.1)	879 (55.5)	0.359	1105 (65.9)	873 (67.2)	0.438
University education	278 (7.4)	106 (6.7)	0.376	140 (8.3)	105 (8.1)	0.798
Employment	173 (4.6)	77 (4.9)	0.668	89 (5.3)	91 (7.0)	0.053
*History of cardiovascular events* ^a^						
History of ischemic stroke	771 (20.5)	294 (18.6)	0.113	217 (12.9)	194 (14.9)	0.116
History of TIA	315 (8.4)	118 (7.4)	0.266	110 (6.6)	102 (7.9)	0.172
History of MI	269 (7.1)	142 (9.0)	**0.022**	83 (4.9)	74 (5.7)	0.364
*Comorbid conditions*						
Number of comorbid conditions	3.0 [2.0]	3.0 [2.0]	0.999 *	3.0 [2.0]	3.0 [2.0]	<0.001 *
Arterial hypertension	3201 (84.9)	1319 (83.3)	0.126	1275 (76.0)	1045 (80.4)	**0.004**
Chronic heart failure	366 (9.7)	190 (12.0)	**0.012**	87 (5.2)	81 (6.2)	0.218
Atrial fibrillation	663 (17.6)	275 (17.4)	0.840	214 (12.8)	178 (13.7)	0.447
Diabetes mellitus	1871 (49.6)	781 (49.3)	0.822	631 (37.6)	527 (40.6)	0.100
Hypercholesterolemia	1866 (49.5)	778 (49.1)	0.793	771 (45.9)	730 (56.2)	**<0.001**
Dementia	304 (8.1)	136 (8.6)	0.527	96 (5.7)	59 (4.5)	0.151
Depression	445 (11.8)	179 (11.3)	0.598	193 (11.5)	172 (13.2)	0.151
Anxiety disorders	1174 (31.1)	491 (31.0)	0.913	513 (30.6)	452 (34.8)	**0.015**
Parkinson’s disease	184 (4.9)	73 (4.6)	0.669	59 (3.5)	46 (3.5)	0.971
Epilepsy	113 (3.0)	47 (3.0)	0.952	37 (2.2)	34 (2.6)	0.465
Bronchial asthma/COPD	878 (23.3)	380 (24.0)	0.584	377 (22.5)	319 (24.6)	0.181
*Statin-related characteristics*						
Initial statin						
Simvastatin	545 (14.5)	268 (16.9)	0.200	195 (11.6)	200 (15.4)	**0.003**
Rosuvastatin	253 (6.7)	107 (6.8)		126 (7.5)	120 (9.2)	
Atorvastatin	2887 (76.6)	1171 (73.9)		1328 (79.1)	947 (72.9)	
Fluvastatin	72 (1.9)	31 (2.0)		21 (1.3)	22 (1.7)	
Lovastatin	12 (0.3)	7 (0.4)		8 (0.5)	10 (0.8)	
New statin user ^b^	312 (8.3)	98 (6.2)	**0.009**	190 (11.3)	105 (8.1)	**0.003**
Intensity of statin treatment ^c^						
Moderate	2847 (75.5)	1120 (70.7)	**0.001**	1321 (78.7)	972 (74.8)	**0.029**
Low	106 (2.8)	51 (3.2)		45 (2.7)	49 (3.8)	
High	816 (21.7)	413 (26.1)		312 (18.6)	278 (21.4)	
Patient´s co-payment (EUR) ^d^	0.7 [1.1]	0.7 [0.8]	0.036 *	0.8 [1.1]	0.7 [1.1]	0.023 *
General practitioner as index prescriber ^e^	2567(68.1)	1039 (65.6)	0.073	1030 (61.4)	778 (59.9)	0.409
*Cardiovascular co-medication*						
Number of medications	10.0 [2.0]	10.0 [3.0]	0.672 *	9.0 [4.0]	10.0 [3.0]	<0.001 *
Number of CV medications	5.0 [3.0]	5.0 [3.0]	0.828 *	5.0 [3.0]	5.0 [4.0]	<0.001 *
Antiplatelet agents	3170 (84.1)	1325 (83.6)	0.677	1333 (79.4)	1051 (80.9)	0.320
Cardiac glycosides	360 (9.6)	149 (9.4)	0.869	85 (5.1)	41 (3.2)	**0.010**
Antiarrhythmic agents	337 (8.9)	155 (9.8)	0.329	102 (6.1)	104 (8.0)	**0.040**
Beta-blockers	839 (22.3)	345 (21.8)	0.699	322 (19.2)	245 (18.9)	0.821
Loop diuretics	1013 (26.9)	438 (27.7)	0.561	265 (15.8)	216 (16.6)	0.539
Mineralocorticoid receptor antagonists	384 (10.2)	171 (10.8)	0.506	76 (4.5)	58 (4.5)	0.933
Anticoagulants	1103 (29.3)	478 (30.2)	0.505	380 (22.6)	319 (24.6)	0.222
Thiazide diuretics	844 (22.4)	364 (23.0)	0.639	338 (20.1)	314 (24.2)	**0.008**
Calcium channel blockers	1216 (32.3)	501 (31.6)	0.650	482 (28.7)	406 (31.3)	0.135
RAAS inhibitors	3328 (88.3)	1384 (87.4)	0.341	1399 (83.4)	1097 (84.4)	0.429
Lipid lowering agents other than statins ^f^	415 (11.0)	160 (10.1)	0.326	166 (9.9)	129 (9.9)	0.973

In case of categorical variables, values represent the frequency, and the percentages are provided in parentheses (% of *n*). In case of continuous variables, medians [interquartile ranges] are provided. TIA—transient ischemic attack; MI—myocardial infarction; COPD—chronic obstructive pulmonary disease; CV—cardiovascular; RAAS—renin-angiotensin-aldosterone-system; *p*—statistical significance between adherent and non-adherent patients according to the χ^2^-test. * Statistical significance according to the Mann–Whitney U test; in case of statistical significance, the values are expressed in bold. ^a^ The time period covered by “history”—5 years before the index date of this study. ^b^ New statin user—patient in whom statin treatment was initiated in association with the diagnosis of peripheral arterial disease (PAD). ^c^ Intensity of statin treatment —low, moderate, high (identified according to dosage per day [11]). ^d^ Co-payment—calculated as the cost of statin treatment paid by the patient per month. ^e^ General practitioner as index prescriber (the follow-up of all patients with PAD is done by angiologists in cooperation with general practitioners). ^f^ Lipid lowering agents other than statins—ezetimibe and fibrates.

**Table 2:** Multivariate analysis of the influence of patient-associated characteristics on the likelihood of non-adherence (*n* = 8330).

Factor	Persistent *n* = 5353	Non-Persistent *n* = 2977
	HR (95% CI)	HR (95% CI)
*Socio-demographic characteristics*		
Age	0.99 (0.98–1.01)	**0.98 (0.97–0.99)**
Female sex	1.07 (0.94–1.22)	0.99 (0.84–1.18)
University education	0.91 (0.71–1.16)	0.90 (0.67–1.20)
Employment	1.07 (0.80–1.43)	**1.41 (1.01–1.96)**
*History of cardiovascular events* ^a^		
History of ischemic stroke	0.89 (0.76–1.05)	1.12 (0.89–1.40)
History of TIA	0.91 (0.72–1.15)	1.11 (0.82–1.49)
History of MI	1.23 (0.98–1.54)	0.99 (0.70–1.39)
*Comorbid conditions*		
Number of comorbid conditions	1.03 (0.88–1.21)	1.21 (0.98–1.48)
Arterial hypertension	0.81 (0.63–1.05)	0.86 (0.64–1.17)
Chronic heart failure	1.27 (0.97–1.66)	0.96 (0.65–1.43)
Atrial fibrillation	0.89 (0.70–1.14)	0.79 (0.56–1.11)
Diabetes mellitus	0.93 (0.76–1.13)	0.85 (0.66–1.10)
Hypercholesterolemia	0.95 (0.77–1.16)	1.11 (0.86–1.43)
Dementia	1.08 (0.82–1.42)	**0.60 (0.40–0.90)**
Depression	0.90 (0.70–1.16)	0.88 (0.64–1.21)
Anxiety disorders	0.98 (0.80–1.21)	0.94 (0.72–1.22)
Parkinson’s disease	0.96 (0.69–1.34)	0.76 (0.48–1.21)
Epilepsy	0.96 (0.65–1.43)	0.95 (0.56–1.64)
Bronchial asthma/COPD	1.0 (0.80–1.24)	0.82 (0.62–1.08)
*Statin-related characteristics*		
Initial statin		
Simvastatin	1.00	1.00
Rosuvastatin	0.87 (0.63–1.20)	0.83 (0.56–1.23)
Atorvastatin	**0.74 (0.62–0.89)**	**0.66 (0.51–0.84)**
Fluvastatin	0.92 (0.58–1.45)	1.02 (0.53–1.98)
Lovastatin	1.44 (0.53–3.92)	1.43 (0.50–4.09)
New statin user ^b^	**0.72 (0.55–0.94)**	0.93 (0.70–1.23)
Intensity of statin treatment ^c^		
Moderate	1.00	1.00
Low	0.92 (0.63–1.36)	0.95 (0.57–1.58)
High	**1.35 (1.16–1.56)**	**1.23 (1.01–1.50)**
Patient´s co-payment (EUR) ^d^	**0.94 (0.88–0.99)**	0.94 (0.88–1.00)
General practitioner as index prescriber ^e^	0.88 (0.78–1.00)	0.95 (0.81–1.11)
*Cardiovascular co-medication*		
Number of medications	0.99 (0.95–1.02)	**1.06 (1.01–1.10)**
Number of CV medications	0.98 (0.93–1.04)	1.06 (0.98–1.14)
Antiplatelet agents	1.01 (0.84–1.20)	0.94 (0.76–1.16)
Cardiac glycosides	0.96 (0.76–1.21)	**0.51 (0.33–0.78)**
Antiarrhythmic agents	1.16 (0.91–1.46)	1.23 (0.87–1.74)
Beta-blockers	0.99 (0.84–1.17)	**0.76 (0.61–0.95)**
Loop diuretics	1.06 (0.88–1.26)	0.92 (0.72–1.18)
Mineralocorticoid receptor antagonists	0.98 (0.78–1.24)	0.87 (0.58–1.29)
Anticoagulants	1.07 (0.91–1.26)	0.89 (0.72–1.11)
Thiazide diuretics	1.08 (0.92–1.27)	1.05 (0.86–1.30)
Calcium channel blockers	1.0 (0.85–1.16)	1.0 (0.82–1.22)
RAAS inhibitors	0.97 (0.79–1.20)	0.89 (0.70–1.13)
Lipid lowering agents other than statins ^f^	0.92 (0.74–1.13)	0.80 (0.61–1.04)

HR—hazard ratio; 95% CI–95% confidence interval. In case of statistical significance (*p* < 0.05), the values are expressed in bold. TIA—transient ischemic attack; MI—myocardial infarction; COPD—chronic obstructive pulmonary disease; CV—cardiovascular; RAAS–renin angiotensin aldosterone system. ^a^ The time period covered by “history”—5 years before the index date of this study. ^b^ New statin user—patient in whom statin treatment was initiated in association with the diagnosis of peripheral arterial disease. ^c^ Intensity of statin treatment—low, moderate, high (identified according to dosage per day [11]). ^d^ Co-payment—calculated as the cost of statin treatment paid by the patient per month. ^e^ General practitioner as index prescriber (the follow-up of all patients with PAD is done by angiologists in cooperation with general practitioners). ^f^ Lipid lowering agents other than statins—ezetimibe and fibrates.

## Supplementary Materials

The following are available online at https://www.mdpi.com/2227-9059/8/10/378/s1, Supplementary Table S1: Multivariate analysis of the influence of patient-associated characteristics on the likelihood of non-adherence evaluated in models using different thresholds defining non-adherence (*n* = 8330); Supplementary Table S2: Multivariate analysis of the influence of patient-associated characteristics on the likelihood of non-adherence evaluated in model with shorter 3-year follow-up period and with standard 80% threshold defining non-adherence (*n* = 8330).
