# Supplementary material for: Non-Adherence to Statin Treatment in Older Patients with Peripheral Arterial Disease Depending on Persistence Status
Source: Biomedicines. 2020 Sep 25;8(10):378. doi: 10.3390/biomedicines8100378 (PMC7599852; doi:10.3390/biomedicines8100378)
Supplement: Supplementary file 1 [file biomedicines-08-00378-s001.zip › Supplementary Table 2.rtf]

Supplementary Table 2 Multivariate analysis of the influence of patient-associated characteristics on the likelihood of non-adherence evaluated in model with shorter 3-year follow-up period and with standard 80% threshold defining non-adherence (n=8330)
Factor	Persistent
n=5353	Non-persistent
n=2977	
	HR (95% CI)	HR (95% CI)	
Socio-demographic characteristics			
Age	1.00 (0.99–1.01)	0.98 (0.97–0.99)	
Female sex	1.11 (0.98–1.26)	1.00 (0.85–1.19)	
University education	0.94 (0.74–1.19)	0.90 (0.68–1.20)	
Employment	1.02 (0.77–1.36)	1.44 (1.03–1.99)	
History of cardiovascular eventsa			
History of ischemic stroke	0.90 (0.77–1.06)	1.02 (0.82–1.28)	
History of TIA	0.92 (0.73–1.15)	0.97 (0.72–1.31)	
History of MI	1.13 (0.90–1.42)	1.03 (0.74–1.45)	
Comorbid conditions			
Number of comorbid conditions	1.01 (0.86–1.18)	1.17 (0.95–1.43)	
Arterial hypertension	0.78 (0.61–0.99)	0.94 (0.70–1.27)	
Chronic heart failure 	1.23 (0.94–1.60)	0.98 (0.66–1.45)	
Atrial fibrillation	1.01 (0.79–1.28)	0.77 (0.55–1.08)	
Diabetes mellitus	0.95 (0.78–1.16)	0.83 (0.64–1.08)	
Hypercholesterolemia	0.82 (0.67–1.01)	1.06 (0.82–1.37)	
Dementia	1.09 (0.83–1.43)	0.59 (0.39–0.88)	
Depression	0.87 (0.68–1.12)	0.96 (0.70–1.31)	
Anxiety disorders	1.05 (0.85–1.29)	0.98 (0.75–1.28)	
Parkinson's disease	0.95 (0.68–1.32)	0.76 (0.48–1.22)	
Epilepsy	0.97 (0.66–1.43)	0.83 (0.48–1.42)	
Bronchial asthma/COPD	1.12 (0.90–1.39)	0.89 (0.67–1.17)	
(Table continued)	
Statin-related characteristics			
Initial statin			
Simvastatin	1.00	1.00	
Rosuvastatin	0.70 (0.51–0.97)	0.74 (0.50–1.09)	
Atorvastatin	0.83 (0.69–0.99)	0.76 (0.59–0.97)	
Fluvastatin	1.00 (0.64–1.57)	0.95 (0.50–1.83)	
Lovastatin	0.81 (0.28–2.40)	1.48 (0.52–4.22)	
New statin userb	0.49 (0.37–0.65)	0.77 (0.58–1.02)	
Intensity of statin treatmentc			
Moderate	1.00	1.00	
Low	0.89 (0.60–1.32)	0.89 (0.53–1.48)	
High	1.37 (1.18–1.59)	1.20 (0.98–1.47)	
Patient´s co-payment (EUR)d	1.01 (0.95–1.07)	1.00 (0.94–1.07)	
General practitioner as an index prescriber	0.81 (0.72–0.92)	0.90 (0.77–1.05)	
Cardiovascular co-medication			
Number of medications 	0.98 (0.94–1.01)	1.04 (0.99–1.08)	
Number of CV medications	0.90 (0.85–0.95)	1.01 (0.93–1.08)	
Antiplatelet agents	1.23 (1.03–1.48)	1.02 (0.82–1.26)	
Cardiac glycosides	1.02 (0.81–1.29)	0.61 (0.40–0.93)	
Antiarrhythmic agents	1.06 (0.84–1.35)	1.20 (0.85–1.69)	
Beta-blockers	1.06 (0.90–1.25)	0.86 (0.69–1.07)	
Loop diuretics	1.24 (1.04–1.48)	0.92 (0.72–1.18)	
Mineralocorticoid receptor antagonists	0.97 (0.77–1.23)	0.85 (0.57–1.27)	
Anticoagulants	1.20 (1.02–1.41)	0.96 (0.77–1.19)	
Thiazide diuretics	1.27 (1.09–1.49)	1.14 (0.92–1.39)	
(Table continued)	
Calcium channel blockers	1.10 (0.95–1.29)	1.00 (0.82–1.22)	
RAAS inhibitors	1.09 (0.88–1.34)	1.01 (0.79–1.28)	
Lipid lowering agents other than statins	1.16 (0.94–1.42)	0.82 (0.63–1.07)	
HR – hazard ratio; 95% CI – 95% confidence interval. In case of statistical significance (p<0.05), the values are expressed in bold. TIA – transient ischemic attack; MI – myocardial infarction; COPD – chronic obstructive pulmonary disease; CV – cardiovascular; RAAS – renin angiotensin aldosterone system.
aThe time period covered by "history" – 5 years before the index date of this study 
bNew statin user – patient in whom statin treatment was initiated in association with PAD diagnosis
cIntensity of statin treatment – low, moderate, high (identified according to dosage per day [11]) 
dCo-payment – calculated as the cost of statin treatment paid by the patient per month
